# Evidence for Enhanced Multisensory Facilitation with Stimulus Relevance: An Electrophysiological Investigation

**DOI:** 10.1371/journal.pone.0052978

**Published:** 2013-01-23

**Authors:** Ayla Barutchu, Dean R. Freestone, Hamish Innes-Brown, David P. Crewther, Sheila G. Crewther

**Affiliations:** 1 School of Psychological Sciences, La Trobe University, Melbourne, Victoria, Australia; 2 The Bionics Institute, Melbourne, Victoria, Australia; 3 The Brain Sciences Institute, Swinburne University, Melbourne, Victoria, Australia; 4 The NeuroEngineering Laboratory, and Electronic Engineering, The University of Melbourne, Melbourne, Victoria, Australia; 5 The Florey Institute of Neuroscience and Mental Health, The University of Melbourne, Melbourne, Victoria, Australia; CNRS - Université Claude Bernard Lyon 1, France

## Abstract

Currently debate exists relating to the interplay between multisensory processes and bottom-up and top-down influences. However, few studies have looked at neural responses to newly paired audiovisual stimuli that differ in their prescribed relevance. For such newly associated audiovisual stimuli, optimal facilitation of motor actions was observed only when both components of the audiovisual stimuli were targets. Relevant auditory stimuli were found to significantly increase the amplitudes of the event-related potentials at the occipital pole during the first 100 ms post-stimulus onset, though this early integration was not predictive of multisensory facilitation. Activity related to multisensory behavioral facilitation was observed approximately 166 ms post-stimulus, at left central and occipital sites. Furthermore, optimal multisensory facilitation was found to be associated with a latency shift of induced oscillations in the beta range (14–30 Hz) at right hemisphere parietal scalp regions. These findings demonstrate the importance of stimulus relevance to multisensory processing by providing the first evidence that the neural processes underlying multisensory integration are modulated by the relevance of the stimuli being combined. We also provide evidence that such facilitation may be mediated by changes in neural synchronization in occipital and centro-parietal neural populations at early and late stages of neural processing that coincided with stimulus selection, and the preparation and initiation of motor action.

## Introduction

Multisensory integration refers to the combination of signals from different sensory systems resulting in behavioral and neural changes that cannot be explained by unisensory function [1]. It is a selective process guided by the temporal and spatial properties of stimuli. Both perceptual sensitivity and motor reaction times are facilitated when stimuli appear in a location cued by another sensory modality [2], [3]. Furthermore, it has been shown that multiple sensory stimuli, with no prior associations, and preceding irrelevant cues can modulate event-related potentials (ERPs) in scalp regions traditionally associated with sensory specific processing [4]–[9]. Indeed Fort et al. [10] also showed that both congruent and incongruent audiovisual objects can modulate neural activity in sensory specific regions following a short learning phase. Collectively, these results suggest that multisensory integration is at least partially determined by bottom-up multisensory inputs.

On the other hand, top-down influences associated with attention, prior knowledge and environmental experience also have the potential to modulate neural processes associated with multisensory integration [10]–[16]. For example, Molholm and colleagues [17] used common objects (i.e., pictures of animals and their associated sounds) to show that the semantic congruence of multisensory stimuli can modulate the visually evoked N_1_ component at occipital-temporal scalp electrodes. More recently, the congruence of looming signals has also been shown to modulate neural activity at post stimulus latencies as early as 75 ms [18]. However, from these studies the effects of stimulus congruence and task related stimulus relevance cannot be dissociated. Indeed, the degree of multisensory facilitation of motor actions has been shown to be much greater for dual auditory and visual targets [19]–[21]. These studies suggest that top-down factors play an important role in the selective integration of multisensory inputs, ensuring that integrative processes are environmentally and functionally relevant to the task at hand. However, the neural mechanism of top-down influences relating to the selection of relevant novel multisensory stimuli remains to be explored.

The synchronous oscillation of neural cell populations has also been implicated in the binding of information both within and between sensory systems [22], and in the top-down modulation of sensory integration [23], [24]. In particular, alpha (8–13 Hz) and beta (13–30 Hz) oscillations are believed to be involved, not only in sensory selection [25] and the preparation and initiation of voluntary motor actions [26]–[29], but also in multisensory integration and its facilitative effect on motor actions [30]. More recently, it has been shown that the phase of local field potentials in the primary auditory cortex of primates is related to the level of response to somatosensory inputs to this region [31], [32]. To our knowledge, no human electrophysiological study has investigated both event-related potentials and whether low-frequency oscillations may be related to multisensory facilitation and top-down processes associated with task specific relevance.

The aim of this study was to investigate the neural mechanisms associated with the selective integration of audiovisual stimuli that results in ‘multisensory motor facilitation’, and how these processes are related to the nominated relevance of the sensory signals. We restricted our study to the investigation of behavioral responses, event-related potentials (ERPs), and induced oscillatory activity in response to audiovisual stimuli with target and irrelevant (i.e., non-target) components. Furthermore, to establish that multisensory motor facilitation was only observed when both audition and vision were targets, accuracy and reaction time measures for both unisensory and audiovisual stimuli were analyzed. To maintain high external validity, sensory stimuli consisted of novel combinations of simple tones and colored flashes of light, since they are commonly used as warning or action signals in man-made objects (e.g., electronic devices, heavy machinery and transport vehicles). Our expectation was that all transient flashes of colored lights or simple sounds would act as alarm or arousal signals. The environmental relevance of red was reversed from the expected ‘stop’ signal to be an action ‘go’ signal, to ensure that participants made an executive decision to respond. We predicted that multisensory facilitation of motor responses would be greatest for audiovisual stimuli with dual relevant targets, and expected integrative processes and stimulus relevance to begin modulating ERPs at scalp electrodes known to be associated with sensory processing. We also expected induced alpha and beta oscillations to be modulated by the relevance of the sensory stimuli being combined.

## Materials and Methods

### Participants

Participants included 14 female and 17 male paid volunteers in the age range of 18 to 31 years (*M* age = 23 years, 6 months; *SD* = 3 years, 3 months) who were right handed, had normal or corrected to normal vision, normal hearing, were not taking any medication at the time of the study and reported having no prior history of neurological or psychiatric disorders. All participants gave written informed consent and all experimental procedures were approved by the La Trobe University Human Ethics Committee.

### Stimuli and Procedure

The electroencephalograph (EEG) was recorded while participants performed an audiovisual discrimination task (i.e., divided attention task). Participants were seated in a dim, sound attenuated room and were asked to visually fixate on a multicolored light-emitting diode attached to the center of a speaker. The speaker was positioned at a distance of 1 m from the participant's eyes, in line with the central point of fixation. Stimulus relevance was manipulated by assigning target and irrelevant (i.e., non-target) stimuli to the auditory and visual modalities. The auditory target (AT) and irrelevant (AI) stimuli were 1000 Hz and 500 Hz pure tones, respectively. All stimuli were presented for 100 ms. Auditory stimuli had a 10 ms onset/offset ramp and were presented at 75 dB SPL measured near the participant's ear. The visual target (VT) and irrelevant (VI) stimuli were red and green flashes, respectively. Visual stimuli were matched for apparent luminance. The auditory (AI and AT) and visual (VI and VT) stimuli were combined to create three irrelevant stimuli: AI, VI and AIVI, and five target stimuli: AT, VT, ATVI, AIVT and ATVT. The presentation order of stimuli throughout the 8 to 10 blocks of 200 stimuli was random. In each block, 25% of the stimuli were targets with an equal probability of presenting AT, VT, ATVI, AIVT or ATVT (i.e., the probability of presentation of each target type was 5%). The remaining 75% of the stimuli were irrelevant with an equal probability of presenting an AI, VI or AIVI stimulus. The inter stimulus-interval (ISI) was randomly varied between 1000 and 1400 ms in steps of 50 ms. Participants were instructed that a ‘target’ stimulus would contain either a red light or a high tone, or both simultaneously. They were asked to attend equally to both auditory and visual stimuli, to press a button with their right index finger if a target stimulus was detected, and to refrain from pressing the button at all other times. All participants were given at least one block of 200 stimuli as practice. Testing commenced once an overall accuracy level above 80% was achieved. The duration of each block of trials ranged from 4 to 5 minutes. Between each block of trials participants were offered a break.

### Analysis of Behavioral Measures

Only motor reaction times (RTs) within the range of 100–800 ms were accepted as correct responses and included in further data analyses. Most participants did not make any errors, and error rates generally violated the assumption of normality. Therefore, non-parametric statistics were applied with Friedman's test being used to compare error rates across the five target stimuli. Significant effects were followed-up with pair-wise comparisons using Wilcoxon signed rank tests.

Mean RTs for the five target stimuli were analyzed using a one-way analysis of variance (ANOVA) followed up with post-hoc test using the Tukey HSD method. The race model prediction of inequality [20] was also tested [33]. In the present study, the cumulative density functions (CDFs) of the unisensory stimuli and the audiovisual stimuli with single targets were summed (AT CDF+VT CDF and AIVT CDF+ATVI CDF). A 3×10 repeated measure ANOVA was applied to compare the RTs of the ATVT, AT+VT, and AIVT+ATVI CDFs across the 10 probability values used to fit the CDFs. For all ANOVAs Greenhouse-Guesser corrections were applied to correct for violations of the assumption of sphericity where appropriate.

### Electrophysiology

Scalp EEG was recorded from 26 sintered Ag/AgCl electrodes attached to a cap: Fp1, Fp2, F7, F3, Fz, F4, F8, FC3, FCz, FC4, T3, C3, Cz, C4, T4, CP3, CPz, CP4, T5, P3, Pz, P4, T6, O1, Oz, and O2. Two additional electrodes were attached to the left and right mastoid (M1 and M2). Horizontal and vertical electro-oculograms were recorded from electrodes positioned above and below the right eye and the outer canthi of both eyes. All recordings were referenced to the nose and re-referenced offline to the common average [34], [35]. Continuous EEG was recorded at a sampling rate of 1 kHz (online band-pass filter: .1–100 Hz, 12 dB/octave). Electrode impedance was maintained below 10 kΩ throughout the recordings. Ocular artefacts were corrected offline using the Gratton and Coles [36] method. Most participants showed continuous muscle related artefacts (i.e., EMG activity) at either one or more of the following electrode sites: T3, T4, F7, F8, Fp1 or Fp2. Due to the high sensitivity of time-frequency analyses to motor artefacts, these electrodes were excluded from all further analyses. All remaining electrodes formed a grid like pattern sampling from frontal to occipital scalp regions. To reduce the effects of volume conduction on neighboring electrodes [37], the remaining scalp electrodes were re-referenced to their common average and the EEG was segmented into epochs of 1400 ms duration (400 ms pre-stimulus and 1000 ms post stimulus). Epochs and scalp regions with gross motor artefacts were identified by visual inspection and removed from further data analyses. Epochs with samples exceeding ±60 µV were also excluded from all further analyses. Over 70% of trials for two participants (one male and one female) were contaminated by artefact and consequently these participants were excluded from further data analyses. Between 40 and 80 trials per stimulus were maintained in the ERP and time-frequency analyses for all remaining participants.

### Event-Related-Potentials (ERPs)

ERPs were derived by baseline correcting each epoch (by subtracting the mean from −200 to 0 ms from the whole epoch), and averaging across epochs for each stimulus type separately. ERPs for ATVT, AIVT and ATVI stimuli were calculated, and analyzed with the assumption that common significant differences between the dual-target ATVT stimulus and both of the audiovisual stimuli with single target components (ATVI and AIVT) were related to the ‘facilitative effect’ of multisensory integration. Conversely, if ERP components for the ATVT stimulus match either or both, AIVT or ATVI it is posited that these components are dependent on the common auditory or visual stimulus properties since ATVT shares one identical stimulus component with each of ATVI and AIVT. Similar conjunction based techniques have previously been used to isolate multisensory processes in fMRI studies [38]. Here we are using the inverse of a conjunction analysis to isolate neural activity related to multisensory motor facilitation only observed for the ATVT stimulus. Indeed this approach has the advantage of identifying neural activity related to the ‘facilitative effect’ of multisensory integration on motor actions without relying on the subtraction of ERP signals. Note that this approach is not sensitive to multisensory neural processes common to all multisensory signals (ATVT, AIVT and ATVI). For an analysis of multisensory integration and a comparison of ERPs in response to unisensory and multisensory stimuli using the subtraction method see Text S1 and Figures S1 and S2.

Statistical analyses were conducted at central, parietal and occipital electrodes, as prior studies have implicated these regions in multisensory processing [39]. For occipital (O1, Oz and O2) and parietal (P3, Pz and P4) electrodes the local peak maxima for the P_1_ (50–180 ms) and P_3_ (200–500 ms) components, and the local peak minima for N_2_ (145–250 ms) component of the ATVT, AIVT and ATVI ERPs were identified. For central electrodes (C3 and C4) the local peak maxima for the P_2_ (150–250 ms) component was also identified. For each component, hemispheric differences in peak amplitude and latency for the three multisensory stimuli at central (C3 and C4), parietal (P3, Pz and P4) and occipital (O1, Oz and O2) electrode sites were analyzed using a series of two-way repeated measures ANOVAs with Greenhouse-Guesser corrections where appropriate. Significant interactions were followed-up with simple effects analyses using the Tukey HSD method.

Measures of the ‘global field power’ (GFP) were also computed, which is equivalent to the standard deviation of all electrodes with respect to the average reference [35]. It has the advantage of being a reference free measure of neural activity, and the disadvantage of providing no information of the source of neural activity. Here we use it to estimate the period when the overall field potential (i.e., signal strength) for the ATVT stimulus deviates from both ATVI and AIVT.

To further assess neural activity related to the facilitative effect of multisensory processing, we employed one-way ANOVAs comparing ATVT, ATVI and AIVT ERPs for each channel and time sample, and for the GFP at each time sample. Significant ANOVAs were followed up with Tukey HSD post-hoc tests. To further control for the inflated Type I error, due to the large number of comparisons, a significant difference was only assumed if ATVT significantly differed from both ATVI and AIVT for 12 consecutive time samples [40].

### Time-Frequency Transformations

The variability of the time-frequency distributions (i.e., power) across trials was calculated to assess the oscillatory fields of the brain in response to the different audiovisual stimuli (ATVT, ATVI and AIVT) [41], [42]. Time-frequency maps were computed using the continuous wavelet transform (Morlet wavelet, parameters *f_c_* = 1, *f_b_* = 1, using the Matlab wavelet toolbox, the MathWorks) with centre frequency *f*, ranging from 8 to 40 Hz in steps of 1 Hz. To isolate induced activity from evoked activity, the mean across trials, *i*, (denoted by 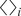 in equation (1)) of the wavelet transformed data, *c*, was subtracted from each respective channel, *α* (with samples indexed by *n*). A measure of the power, *y*, was then obtained as described below.
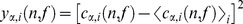
(1)By averaging the band power, *y*, over trials, a feature that is representative of the frequency specific variance over all trials was obtained for each channel by
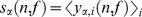
(2)Thus *s* is a measure of inter-trial variance of the time-frequency map. The final processing step was to normalize the inter-trial variance using a background period, *R*, of 150 ms (ranging from −200 to −50 ms relative to stimulus onset, i.e., baseline correction). The normalization (i.e., baseline correction) enables direct comparison between participants and stimuli.
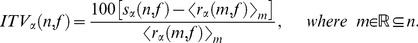
(3)Multiplying by 100 gives the inter-trial variance as a percentage increase or decrease relative to the pre-stimulus reference period. This feature of the evoked-oscillatory activity is also known as event-related desynchronisation/synchronisation (ERD/S) [41], [42], and percentage change in power.

### Time-Frequency Analyses

To exclude distortions produced by the time-frequency transforms at the beginning and end of epochs, follow-up statistical analyses were concentrated in the time range of −200 ms pre-stimulus to 800 ms post-stimulus onset, thus, trimming 200 ms off either end. Significant increases and decreases in inter-trial variance from the baseline across participants for each stimulus type were isolated using a bootstrap procedure. This analysis was only carried out as a first stage exploratory analysis to confirm that changes in inter-trial variance differed from the baseline. For each frequency and time point, 10,000 bootstraps (*B*) were sampled with replacement from the 29 participants. To maintain some control over the inflated Type I error, we employed a two-tailed test with a significance level of α = .01. Lower and upper bound confidence intervals (*CI_LB_* and *CI_UB_*, respectively) were determined using the percentile procedure [43]:
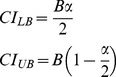
(4)As measures of inter-trial variance were baseline corrected, with zero representing no change in inter-trial variance from pre-stimulus onset, a significant increase in inter-trial variance was assumed if both *CI*'s were positive, and a significant decrease in inter-trial variance was assumed if both *CI*'s were negative. Non-significant differences were occluded from the time-frequency plots using a white mask.

For the alpha (8–13 Hz) and beta (14–30 Hz) frequency bands, data were collapsed across frequencies by averaging. For both alpha and beta bands, the amplitudes and latencies of the local minima between 200–800 ms were identified. For the central electrodes Cz and CPz one-way repeated measures ANOVAs were used to assess significant differences in the amplitudes and the latencies of peak minima across ATVT, AIVT and ATVI stimuli. Hemispheric differences at parietal and occipital sites were further analyzed using 2 (right and left hemisphere)×3 (stimulus) repeated measures ANOVAs. Four two-way ANOVAs were employed to compare differences in the amplitudes and the latencies of peak minima in alpha and beta bands separately. The above bootstrap procedure was also applied to compare the three audiovisual stimuli at each frequency and time sample. To control for the inflated Type I error due to the large number of comparisons, a significant difference was only assumed if ATVT significantly differed from both ATVI and AIVT for 12 consecutive time samples [40].

## Results

### Multisensory Facilitation of Motor Reaction Times and Accuracy

**Figure 1:**
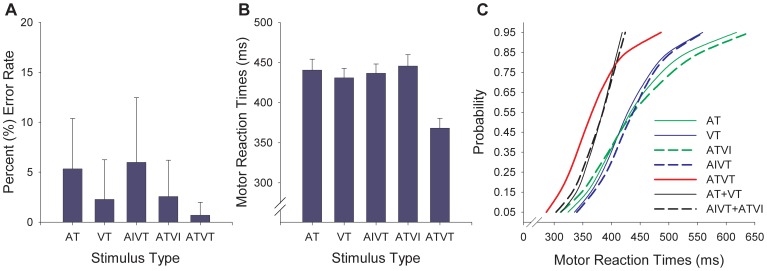
Behavioral measures of multisensory facilitation. **A**. Percent (%) error rate (+*SD*) for unisensory targets (AT and VT) and audiovisual stimuli with target and irrelevant components: ATVI (auditory target and visual irrelevant), AIVT (auditory irrelevant and visual target) and ATVT (audiovisual dual targets). **B**. Mean motor reaction times (+*SEM*) for unisensory (AT and VT) and multisensory stimuli (ATVI, AIVT and ATVT). **C**. Cumulative density functions (CDFs) of motor reaction times (RTs) for AT, VT, ATVI, AIVT, ATVT stimuli, the summed CDFs for unisensory stimuli (AT+VT) and multisensory stimuli with single target components (AIVT+ATVI).

Error rates for the invalid stimuli were very low violating the assumptions of normality, therefore, they were not subjected to further data analyses: AI (*M* = 0.24, *SD* = 0.31), VI (*M* = 0.22, *SD* = 0.34), and AIVI (*M* = 0.51, *SD* = 0.40). For target stimuli, multisensory facilitation of motor response accuracy was observed only for ATVT stimuli, where both audition and vision were targets (see Figure 1A). Fewer errors were made for ATVT stimuli compared to AT, VT, AIVT and ATVI stimuli [χ^2^(4, *N* = 29) = 54.83, *p*<.001]. Multisensory facilitation of RTs was also observed (Figure 1B and 1C). A one-way ANOVA showed that mean RTs (Figure 1B) were significantly faster for ATVT than all other target stimuli (AT, VT, AIVT and ATVI), *F*_2.31, 64.54_ = 80.41, *p*<.001, *η* ^2^ = .74. In addition, a 3 (ATVT CDF, AT+VT CDF, and ATVI+AIVT CDF)×10 (probabilities use to fit CDFs) ANOVA revealed that the ATVT CDF was significantly faster than both the AT+VT CDF and the ATVI+AIVT CDF for values .05 to .65 probability (Figure 1C), *F*_1.65, 46.14_ = 64.29, *p*<.001, *η* ^2^ = .70. Further behavioral testing revealed that these effects are not specific to the combination of colors and tones employed in this study [21]. Similar patterns of behavioral results were observed when different visual (other colors and achromatic objects) and auditory (other pure and complex tones) stimuli were employed.

### Multisensory Facilitation in Event-Related-Potentials

**Figure 2:**
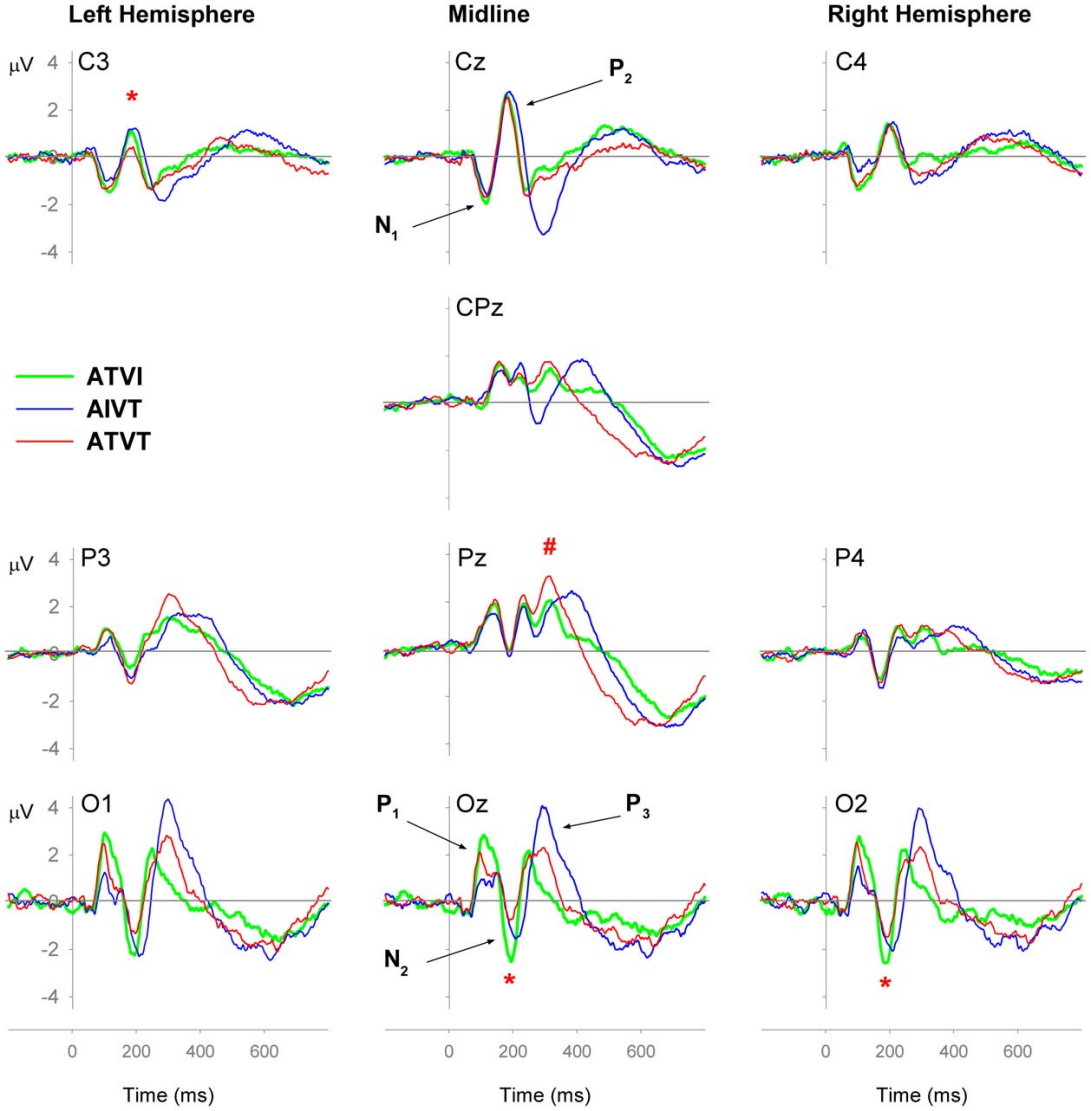
Event-related potentials (ERPs) for audiovisual stimuli with irrelevant components and dual targets. ERPs for the audiovisual stimuli ATVI (auditory target and visual irrelevant), AIVT (auditory irrelevant and visual target) and ATVT (audiovisual dual targets) at central (C3 Cz, C4 and CPz), occipital (O1, Oz and O2) and parietal (P3, Pz and P4) electrode sites. * depicts post-hoc outcomes following a significant interaction effect for voltage difference where ATVT is significantly different from both ATVI and AIVT. # depicts a significant main effect for stimulus type where ATVT is significantly different from both ATVI and AIVT.

**Figure 3:**
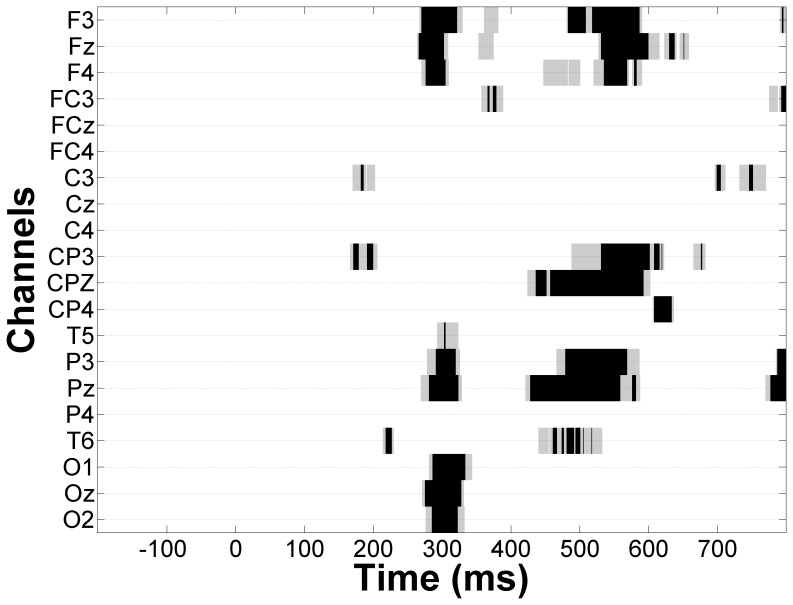
Event-related potential (ERP) components that significantly differ for dual targets. Plot of Q-values from Tukey post-hocs comparisons following significant one-way ANOVAs for ATVI (auditory relevant target and visual irrelevant), AIVT (auditory irrelevant and visual relevant target) and ATVT (audiovisual dual relevant targets) ERPs at each time sample and channel. Shaded regions depict when the ERP for ATVT was significantly different from both, ATVI and AIVT ERPs for 12 consecutive samples. White regions *p*>.05, grey regions *p*<.05 and black regions *p*<.01.

The ERPs for the three audiovisual stimuli with different combinations of relevant target components had similar morphology for the first 300 ms (see Figure 2 for ERPs). The relevance of audiovisual stimuli modulated early activity, with auditory targets (ATVI) leading to greater positive amplitudes of the occipital P_1_ component (see Figure 2, Electrodes O1, Oz and O2). A 3(ATVT, AIVT and ATVI)×3(O1, Oz and O2 electrodes) ANOVA showed a significant main effect for stimulus type (*F*_2, 56_ = 6.06, *p* = .004, *η* ^2^ = .18), with ATVT and ATVI stimuli resulting in significantly greater amplitudes than AIVT (note that this early increase in amplitude may be determined by the relevance of the auditory signal or differences in stimulus properties: 1000 Hz for both ATVT and ATVI vs. 500 Hz for AIVT stimulus). The first notable multisensory facilitative effect, where responses to ATVT stimuli differed from both ATVI and AIVT stimuli, was apparent in the P_2_ component at the left hemisphere electrode C3, but not at the contralateral C4 electrode; a significant interaction was observed between stimulus type (ATVT, AIVT and ATVI) and electrode (C3 and C4), *F*_2, 56_ = 3.74, *p*<.001 (*η* ^2^ = .12). The ATVT ERP began to significantly deviate from both the ATVI and AIVT ERPs at a latency of 166 ms at left hemisphere central-parietal electrodes (Figure 3). Similarly, the negativity observed around the N_2_ component at occipital Oz electrodes was also reduced for ATVT stimuli compared with both ATVI and AIVT stimuli at Oz and O2, but not the O1 electrode, *F*_4, 112_ = 2.60, *p*<.05 (*η* ^2^ = .09). The N_2_ component at occipital sites is a relatively narrow peak and the observed significance is not maintained for 12 consecutive samples (Figure 3). The P_3_ amplitude was also modulated by the relevance of multisensory stimuli with a 3(ATVT, ATVI and AIVT)×3(P3, Pz and P4) ANOVA showing significantly greater amplitudes at the parietal electrodes for ATVT stimuli than both ATVI and AIVT stimuli, *F*_2, 56_ = 15.95, *p*<.001 (*η* ^2^ = .36). Component peak latencies for ATVT and ATVI ERPs did not significantly differ, but both peaked significantly earlier than the AIVT ERP at the occipital P_1_ (*F*_2, 56_ = 5.08, *p* = .009, *η* ^2^ = .15) and N_2_ components (*F*_1.46, 40.92_ = 8.81, *p* = .001, *η* ^2^ = .26), and at the parietal P_3_ component (*F*_2, 56_ = 9.05, *p*<.001 (*η* ^2^ = .24). These early latency shifts are most likely related to differences in auditory stimulus properties with earlier peak for 1000 Hz (ATVT and ATVI stimuli) then 500 Hz (AIVT stimuli). Note that latency shifts where ATVT is different from both the ATVI and AIVT ERPs were only evident approximately 400 ms post-stimulus (see Figure 2 parietal electrodes).

**Figure 4:**
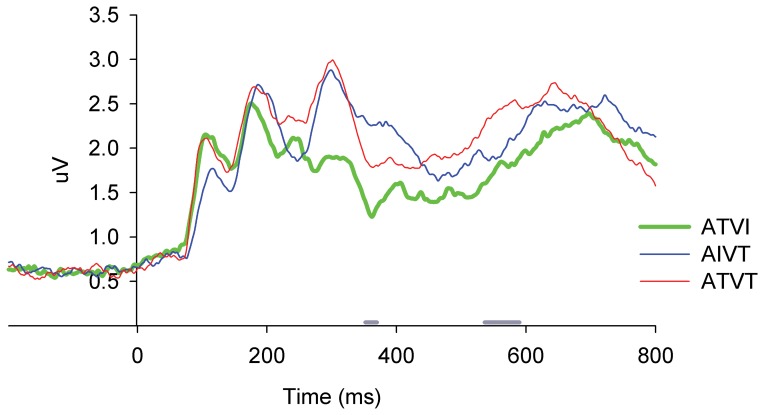
Global Field Power (GDF) and regions that significantly differ for dual targets. GDF measures for ATVI (auditory relevant target and visual irrelevant), AIVT (auditory irrelevant and visual relevant target) and ATVT (audiovisual dual relevant targets) stimuli. Gray bars depict time samples where ATVT significantly differ from both ATVI and AIVT for at least 12 consecutive samples (plot of Q-values from Tukey post-hocs comparisons following significant one-way ANOVAs).

Figure 4 shows global field power (GFP) measures for the three multisensory stimuli. The GDF for ATVT began to significantly differ from ATVI and AIVT at approximately 350 ms post stimulus; The effects of ATVT appear to be come global across the neural network around 350 ms post stimulus onset.

### Time-Frequency Analysis of Oscillatory Activity

**Figure 5:**
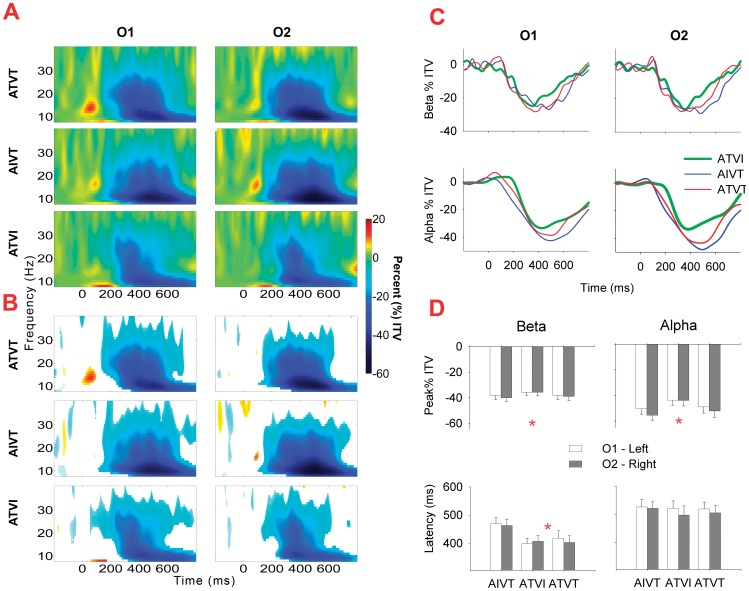
Inter-trial variance for audiovisual stimuli at occipital electrode sites. **A**. For occipital electrodes O1 and O2, percentage (%) of increase and decrease in inter-trial variance (ITV) in the 8–40 Hz frequency range for ATVI (auditory target and visual irrelevant), AIVT (auditory irrelevant and visual target) and ATVT (audiovisual dual target) stimuli. **B**. For each stimulus type at O1 and O2, non-significant (*p*>.01 determined using a bootstrap procedure) changes in inter-trial variability (ITV) from the baseline are occluded using a white mask. **C**. Mean alpha (8–13 Hz) and beta (14–30 Hz) ITV across time for electrodes O1 and O2. **D**. Mean amplitude and latency of peak minima (+*SEMs*) in the alpha and beta frequency range for O1 and O2 electrodes, (* *p*<.05 for main effect of stimulus type for two-way ANOVA).

**Figure 6:**
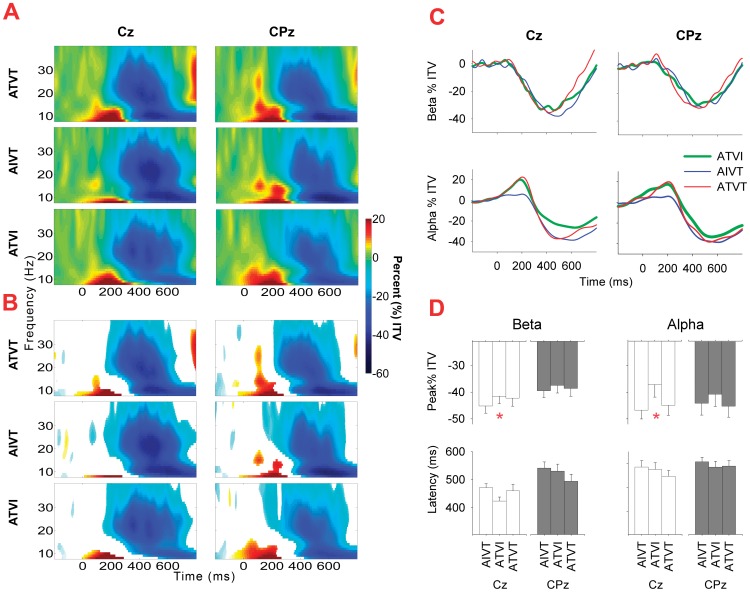
Inter-trial variance for audiovisual stimuli at central electrode sites. **A**. For the central electrode sites Cz and CPz, percentage (%) of increase and decrease in inter-trial variance (ITV) in the 8–40 Hz frequency range for ATVI (auditory target and visual irrelevant), AIVT (auditory irrelevant and visual target) and ATVT (audiovisual dual target) stimuli. **B**. For each stimulus type at Cz and CPz, non-significant (*p*>.01 determined using a bootstrap procedure) changes in inter-trial variance (ITV) from the baseline are occluded using a white mask. **C**. Mean alpha (8–13 Hz) and beta (14–30 Hz) inter-trial variance (ITV) across time for electrodes Cz and CPz. **D**. Mean amplitude and latency of peak minima (+*SEMs*) in the alpha and beta frequency range for Cz and CPz electrodes (* *p*<.05 for one-way ANOVAs).

The mean change in inter-trial variance in the alpha and beta range across participants for the audiovisual stimuli ATVI, AIVT and ATVT are presented in Figures 3, 4, 5. In the alpha (8–13 Hz) and beta (14–30 Hz) bands, localized increases in inter-trial variance were found at occipital (Figure 5) and central (Figure 6) electrodes. All three audiovisual stimuli showed increases in inter-trial variance at the central electrodes Cz and CPz in the alpha range between 120–230 ms (Figure 6), yet only ATVT stimuli showed significant increases in the high beta range (20–30 Hz) at CPz, peaking at a latency of 110 ms (Figure 6B). Similarly for ATVT, early significant increases in the beta frequency range, peaking at ∼60 ms post stimulus onset, at electrode O1 were also observed (Figure 5B), but differences between ATVT and both ATVI and AIVT stimuli did not reach significance.

Late decreases in inter-trial variability in the alpha and beta ranges were distributed with their local peak minima significantly differing not only across the stimuli but also at different scalp locations. The negativity of the peak minima was significantly greater for stimuli with a visual target component (ATVT and AIVT) compared with the ATVI stimulus, where the visual component was irrelevant. This was consistent for inter-trial variance in the alpha band at the central electrode Cz (*F*_1.60, 44.73_ = 7.20, *p* = .004,*η* ^2^ = .21), occipital (*F*_2, 56_ = 8.48, *p* = .001,*η* ^2^ = .23) and parietal (*F*_2, 56_ = 4.42, *p* = .02,*η* ^2^ = .02) sites, and the beta band at parietal sites (*F*_2, 56_ = 8.10, *p* = .001,*η* ^2^ = .22).

**Figure 7:**
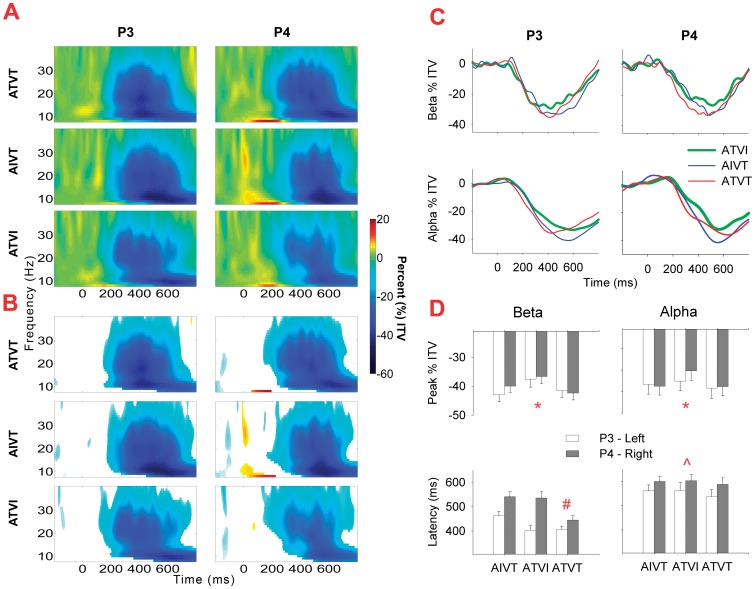
Inter-trial variance for audiovisual stimuli at parietal electrode sites. **A**. For the parietal electrode sites P3 and P4, percentage (%) of increase and decrease in inter-trial variance (ITV) in the 8–60 Hz frequency range for ATVI (auditory target and visual irrelevant), AIVT (auditory irrelevant and visual target) and ATVT (audiovisual dual target) stimuli at the parietal electrode sites P3 and P4. **B**. For each stimulus type at P3 and P4, non-significant (p>.01 determined using a bootstrap procedure) changes in ITV from the baseline are occluded using a white mask. **C**. Mean alpha (8–13 Hz) and beta (14–30 Hz) inter-trial variance (ITV) across time for electrodes P3 and P4. **D**. Mean amplitude and latency of peak minima (+SEMs) in the alpha and beta frequency range for P3 and P4 electrodes (*, ∧ and # *p*<.05 for main effect of stimulus type, hemisphere and the interaction between stimulus type and hemisphere, respectively for the two-way ANOVA).

**Figure 8:**
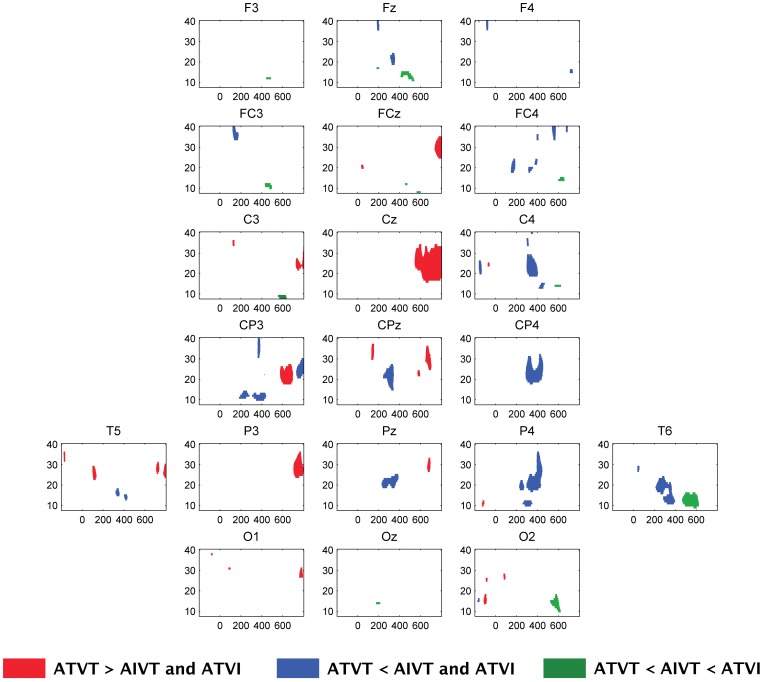
Time-frequency maps of showing areas related to multisensory facilitation. Time-frequency maps depicting regions where the ATVT stimulus was significantly different from both the ATVI and AIVT stimulus. Significant differences were identified using a bootstrap procedure with alpha set at .01.

The latency of peak minima was also affected by stimulus relevance. At occipital electrodes, inter-trial variance in beta band peaked at ∼400 ms for stimuli with auditory targets (ATVI and ATVT), which was earlier than irrelevant auditory stimuli (AIVT), (*F*_2, 56_ = 4.68,*p* = .01, *η* ^2^ = .14). At parietal electrodes, beta band peak latency for the different audiovisual stimuli significantly interacted with hemispheric differences (*F*_2, 56_ = 3.74, *p* = .03, *η* ^2^ = .12). For audiovisual stimuli with irrelevant components, the amplitude at the left hemisphere P3 electrode peaked at a shorter latency than at the right hemisphere P4 electrode (Figure 7C). In contrast, for dual target audiovisual stimuli, both the right and left hemisphere inter-trial variance in the beta band peaked at a similar latency, indicating temporal facilitation of right hemisphere neural responses. Although this effect peaked at approximately 450 ms post stimulus (Figure 7D), responses to ATVT significantly differed from both ATVI and AIVT beginning at approximately 200 ms post stimulus onset lateralized to right hemisphere centro-parietal electrodes (see Figure 8).

## Discussion

Multisensory facilitation of motor reaction times and accuracy was optimal when both auditory and visual stimuli were targets. In the present study, facilitative neural activity was assumed to have occurred when responses to dual target audiovisual stimuli (ATVT) significantly deviated from both audiovisual stimuli with irrelevant non-target components (ATVI and AIVT). Although relevant auditory signals increased ERP amplitudes within 100 ms at occipital sites, this increase was not specific to behavioral multisensory facilitation. In both ERPs and induced oscillations, neural activity specific to dual target multisensory facilitation was first observed after 166 ms post stimulus onset, suggesting that both early and late neural processes contribute to facilitative effects of multisensory integration on motor responses.

### Evidence of Multisensory Facilitation and Relevance in Event-Related-Potentials

Neural processes and behavioral responses associated with audiovisual facilitation have previously been shown to be affected not only by linguistic or semantic congruence [17], [44], [45], but also by the task specific relevance of the stimuli being combined [6], [10]. The findings of the present study further highlight the importance of newly assigned stimulus relevance for optimal multisensory facilitation. All multisensory target stimuli were presented with an equal probability of .05, yet multisensory facilitation of behavioral responses was only observed for stimuli with dual multisensory targets, therefore, this enhancement in accuracy and motor speed cannot be attributed to a pop-out or oddball effect. The relevance of multisensory stimuli modulated neural activity during early sensory processing, and this effect was most pronounced at occipital electrodes. Other electrophysiological studies have suggested that multisensory integration may be initiated within 60 ms or earlier within sensory specific sites [7]–[10], [18], [46]. Consistent with these reports we observed that P_1_ amplitude associated with auditory targets was increased at occipital electrodes compared to auditory non-targets. This early amplitude modulation may be driven by differences in auditory signal properties with the target being a higher frequency than the irrelevant signal. Alternatively, newly acquired knowledge of the relevance of auditory signals may have affect attention processes, and altered the tuning and responsiveness of neural cell populations within the primary visual cortex. Multisensory integration and top-down influences associated with attention have been previously shown to modulate neural activity in parietal and visual brain regions in anticipation of stimuli prior to onset [47], [48], and can be disrupted using trans-cranial magnetic stimulation (TMS) and trans-cranial direct current stimulation (tDCS) [49], [50]. In the present study, given that neural activity was modulated from the initiation of the first visual ERP component (i.e., onset of the visual P_1_ component), top-down inputs may have tuned the visual cortex to anticipate and differentiate relevant auditory stimuli during very early sensory processing. However, this early top-down modulation was not the sole determinant of the motor action facilitation. Early integrative processes can be vetoed or inhibited to further optimize the selectivity of multisensory facilitation depending on the relevance of the stimulus.

In the present study the first differences in ERPs associated with multisensory facilitation were suppressive at left central and occipital scalp regions initiating around 166 ms post stimulus at the P_2_ component, which is generally associated with attention and stimulus selection processes. The later P_3_ component, generally associated with stimulus novelty, memory and attention mechanisms [51], [52], was also modulated by stimulus relevance. At approximate 350 ms changes in global field power were also observed suggesting that by this time the effects of multisensory facilitation generalize across the neural network, with earlier multisensory facilitation effects being highly localized. Semantic congruency of audiovisual objects, operationalized using animal pictures and vocalizations has previously been shown to enhance the N_1_ negativity [17]. This has been further examined by Puce and colleagues who also showed that the N_1_ component elicited by congruent and incongruent audiovisual stimuli is modulated by experience or, as the authors proposed, social relevance [14]. Other studies have reported greater negative deflections after 200 ms post-stimulus onset to irrelevant audiovisual stimuli without pre-existing associations [6]. Incongruent speech stimuli [45], [53]–[55], with easily recognizable audiovisual incongruence have also been reported to induce greater negativity than matching stimuli [55]. Consistent with this previous finding [55], the target and irrelevant non-target sensory stimuli employed in the present study were also easily distinguishable, and audiovisual stimuli with irrelevant components yielded greater N_2_ and P_2_ deflections at occipital and central electrodes than dual target conditions. When sensory stimuli are easily distinguished, incongruence related to the relevance of stimuli may engage more neural activity to dissociate conflicts in ‘go’ and ‘stop’ signals.

The particular associations assigned to our flashes and tones were novel and required reversal of previous environmental learning, but were still easily classified semantically as relevant targets or irrelevant non-targets. Therefore, we hypothesize that the observed changes at left occipital and central scalp regions are representative of neural synchronizations in audiovisual association cortices, such as the superior temporal sulcus (STS). Recently, images of faces coupled with vocalizations have been shown to increase the neural synchronization and oscillatory phase locking between the auditory cortex and the STS in the primate cortex [56]. This increased coherence through phase resetting of neural oscillations between the STS and primary sensory regions has also been implicated in rapid stimulus selection and the inhibition of integrative processes to distracter or irrelevant stimuli [31], [32], [56]. Thus, it is plausible to assume that the altered evoked (i.e., phase-locked) P_2_ component over central and occipital electrodes is representative of changes in neural synchronization and phase locking between auditory and STS neural networks. Alternatively, the left hemisphere lateralization of both early and late P_3_ components at central and parietal electrodes may be related to the fact that all motor responses to relevant target stimuli were made with the right index finger. As highlighted above, the consensus regarding enhancement of the N_2_ and the P_3_ is that such enhancements are generally considered to be associated with stimulus novelty, selection and attention processes [51], [52], suggesting that these effects are not purely motor related and that higher order attention and decision processes are also likely to be influenced by the relevance of audiovisual stimuli. Since ERPs are believed to be representative of the level of neural synchronization to a given stimulus, multiple distributed brain regions would be expected to operate in concert to unify the audiovisual percept at various stages of neural processing.

### Induced Low-Frequency Synchronizations to Relevant Stimuli

Induced neural changes (non-phase locked) in the alpha and beta ranges were also affected by the relevance of sensory stimuli. Early increases in inter-trial variance in alpha and beta bands, which have previously been associated with multisensory integration [57] and the facilitation of motor responses [30], were localized to central and occipital electrodes and were followed by widely distributed decreases in inter-trial variance. Prior studies have shown induced alpha oscillations to be modulated by unisensory target stimuli [58], [59], while decreases in both alpha and beta inter-trial variance are generally associated with visual stimulation [60], [61], and the execution of voluntary motor movements [62]. As the inter-trial variance in both frequency bands showed greater decreases for audiovisual stimuli with visual targets, our findings are consistent with these prior studies in showing that alpha and beta oscillations are not only motor related but are also affected by factors related to visual target selection.

The latency of changes in inter-trial variance in the beta band was also affected by the relevance of audiovisual stimuli. At central-parietal electrodes, beta band oscillations in the left hemisphere preceded those in the right hemisphere only for audiovisual stimuli with irrelevant components, while for stimuli that gave rise to multisensory facilitation, right and left hemisphere beta oscillations were temporally aligned. To our knowledge no prior study has reported such an effect, presumably due to different analysis techniques. For example, most topographic representations of oscillatory activity focus on changes in amplitude of inter-trial variance (i.e., power or ERD/S), and in this case, it is only the latencies of beta oscillations that are affected. This effect also appears to be localized to the parietal electrodes, whereas many previous studies have collapsed electrode activity into regions of interest, which has the potential to smear the effect. Lastly, similar activity in both hemispheres appears after 200 ms post stimulus onset, a time range often not considered when multisensory and unisensory stimuli are contrasted using the subtraction method. The bilateral modulation of motor regions in such tasks is not surprising given that both contralateral and ipsilateral primary and supplementary motor areas are involved in the preparation and performance of voluntary movements as demonstrated by fMRI [63]–[66], TMS [67] and magnetoencephalography [68] studies. It is plausible to suggest that simultaneously engaging contralateral and ipsilateral cortical regions involved in motor preparation and initiation may further enhance motor performance.

## Conclusion

Multisensory facilitation of accuracy and reaction time to newly learnt audiovisual associations was observed to be optimal when both auditory and visual components of the stimuli were relevant to the task at hand. ERPs in response to audiovisual stimuli indicate that multisensory facilitation may be associated with increased phase locking of left hemisphere neural assemblies, especially at central-parietal sites. Furthermore, induced beta oscillations at right and left hemisphere occipital electrodes peaked at a similar time for audiovisual stimuli that gave rise to the facilitation of motor actions. Thus, both ERPs and induced oscillations were modulated by stimulus relevance at a late stage of neural processing, which coincided with preparation and initiation of motor action. However, given the low temporal resolution of time-frequency transforms, particularly for low frequency oscillations in the alpha and beta range [69], our results raise a further fundamental question of whether late oscillations, after 200 ms post stimulus, are driving multisensory facilitation or whether they are a consequence of earlier integrative processes. Our results suggest that the neural synchronization driving behavioral multisensory facilitation may not only involve early processes, within the first 170 ms in sensory specific regions, but also the late activation of neural networks and assemblies engaged in stimulus selection, decision making, and the preparation and initiation of motor actions.

## Supporting Information

Figure S1 **Event-related potentials (ERPs) for irrelevant audiovisual stimuli.** **A**. ERPs for the audiovisual irrelevant stimuli AIVI (auditory irrelevant and visual irrelevant), and the sum of its unisensory components AI (auditory irrelevant) and VI (visual irrelevant) (AI+VI) for occipital (O1 and O2) and parietal (P3 and P4) electrodes. **B**. Plot of significantly different t-tests for each time sample and channel where the AIVI and (AI+VI) ERPs significantly differ for 12 consecutive samples. Shaded regions depicting where the AIVI is significantly different from AI+VI. White regions *p*>.05, grey regions *p*<.05 and black regions *p*<.01.
(TIF)

Figure S2 **Event-related potentials (ERPs) for target audiovisual stimuli.** **A**. ERPs for the audiovisual stimuli ATVT (auditory target and visual target), and the sum of its unisensory components AT (auditory target) and VT (visual target) (AT+VT) for occipital (O1 and O2) and parietal (P3 and P4) electrodes. **B**. Plot of significantly different t-tests for each time sample and channel where the ATVT and (AT+VT) ERPs significantly differ for 12 consecutive samples. Shaded regions depicting where the ATVT ERP is significantly different from the AT+VT ERP. White regions *p*>.05, grey regions *p*<.05 and black regions *p*<.01.
(TIF)

Text S1 **Subtracting audiovisual integration from target and irrelevant stimuli.** Audiovisual integration as assessed using the subtraction method for irrelevant [MSI = AIVI−(AI+VI)] and target stimuli [MSI = ATVT−(AT+VT)].
(DOCX)
