# Supplementary figures and images for: Evidence for Enhanced Multisensory Facilitation with Stimulus Relevance: An Electrophysiological Investigation
Source: PLoS One. 2013 Jan 23;8(1):e52978. doi: 10.1371/journal.pone.0052978 (PMC3553102; doi:10.1371/journal.pone.0052978)

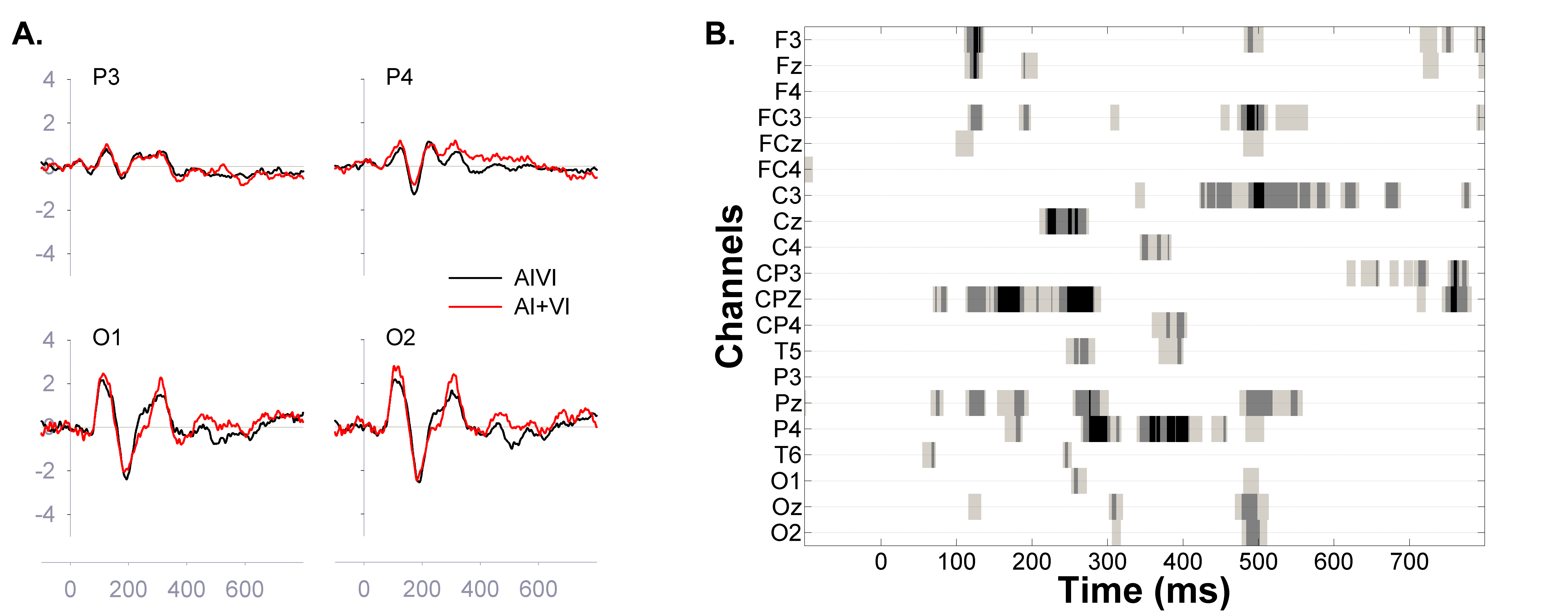

Supplement: Figure S1 — Event-related potentials (ERPs) for irrelevant audiovisual stimuli. A. ERPs for the audiovisual irrelevant stimuli AIVI (auditory irrelevant and visual irrelevant), and the sum of its unisensory components AI (auditory irrelevant) and VI (visual irrelevant) (AI+VI) for occipital (O1 and O2) and parietal (P3 and P4) electrodes. B. Plot of significantly different t-tests for each time sample and channel where the AIVI and (AI+VI) ERPs significantly differ for 12 consecutive samples. Shaded regions depicting where the AIVI is significantly different from AI+VI. White regions p>.05, grey regions p<.05 and black regions p<.01. (TIF) [file pone.0052978.s001.tif]

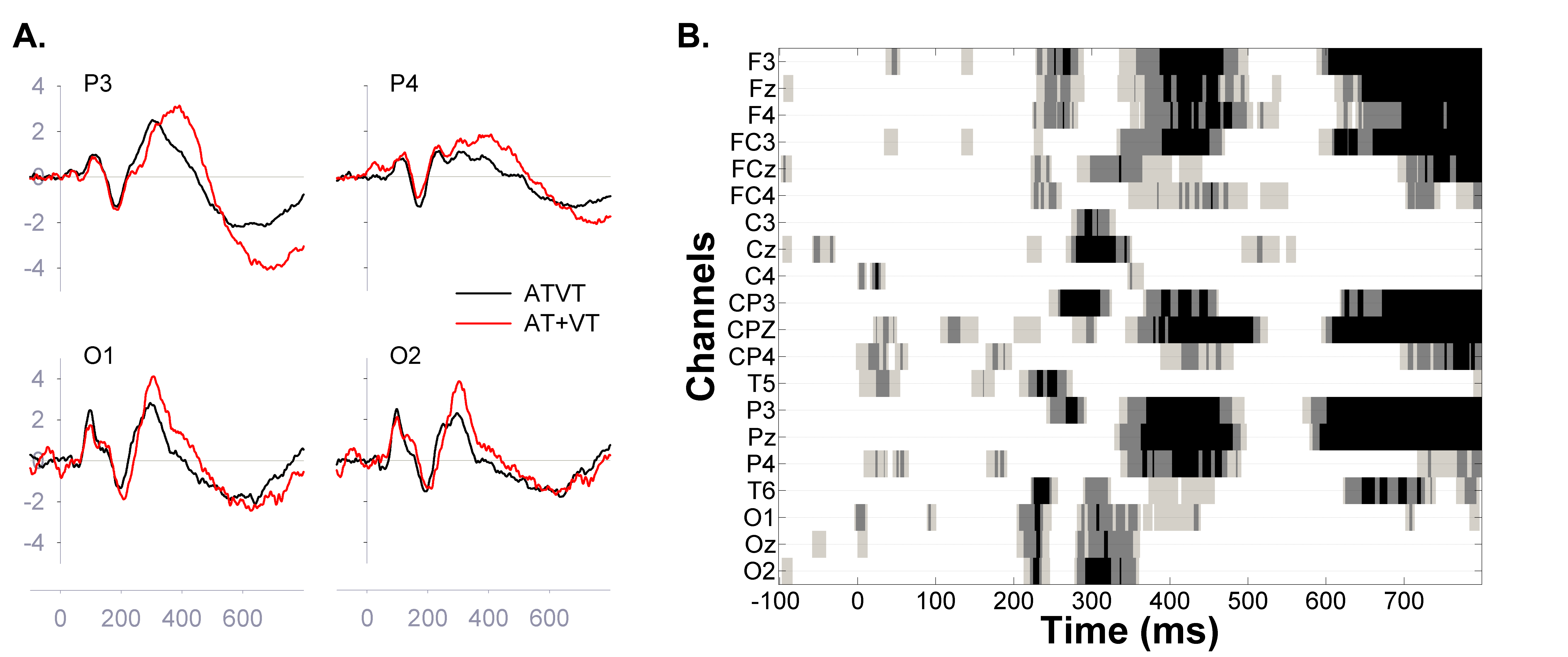

Supplement: Figure S2 — Event-related potentials (ERPs) for target audiovisual stimuli. A. ERPs for the audiovisual stimuli ATVT (auditory target and visual target), and the sum of its unisensory components AT (auditory target) and VT (visual target) (AT+VT) for occipital (O1 and O2) and parietal (P3 and P4) electrodes. B. Plot of significantly different t-tests for each time sample and channel where the ATVT and (AT+VT) ERPs significantly differ for 12 consecutive samples. Shaded regions depicting where the ATVT ERP is significantly different from the AT+VT ERP. White regions p>.05, grey regions p<.05 and black regions p<.01. (TIF) [file pone.0052978.s002.tif]
