# Supplementary material for: Evidence for Enhanced Multisensory Facilitation with Stimulus Relevance: An Electrophysiological Investigation
Source: PLoS One. 2013 Jan 23;8(1):e52978. doi: 10.1371/journal.pone.0052978 (PMC3553102; doi:10.1371/journal.pone.0052978)
Supplement: Text S1 — Subtracting audiovisual integration from target and irrelevant stimuli. Audiovisual integration as assessed using the subtraction method for irrelevant [MSI = AIVI−(AI+VI)] and target stimuli [MSI = ATVT−(AT+VT)]. (DOCX) [file pone.0052978.s003.docx]

**Supplementary Information**

The most common, yet contentious, methods for assessing multisensory processes at a neural level are those that employ subtraction [[1-3](#_ENREF_1)], where multisensory stimuli are compared to the sum of their unisensory counterparts [i.e., multisensory integration MSI = ATVT – (AT + VT)]. Currently, there are no better alternatives for assessing integration in multisensory relative to unisensory ERPs. We have, therefore, applied the subtractive method to assess integrative processes in ERPs evoked by irrelevant (AIVI, AI and VI) and target (ATVT, AT and VT) stimuli (note that time-frequency transforms are not linear, therefore, not included in this analysis). Consistent with prior reports, both irrelevant and target stimuli resulted in significant multisensory integration lateralised to right hemisphere parietal-temporal regions at early latencies before 100 ms post stimulus (Figure S1 and S2) (note that significant differences early, or prior to baseline, are common with the subtraction method, and many studies do not show baseline comparisons) [[4-6](#_ENREF_4)]. Multisensory integration is amplified for target relative to irrelevant stimuli at both early and late latencies. Early differences in multisensory integration between irrelevant and target stimuli are likely to be related to the relevance of stimuli prescribed by the task. The experiment employed an odd-ball paradigm whereby irrelevant and target stimuli were presented at .75 and .25 probability, respectively. Multisensory integration processes may be inhibited to common irrelevant stimuli with integrative processes enhanced to novel targets. One limitation with this comparison is that one cannot dissociate multisensory integration related to stimulus novelty from task relevance. Indeed late subtractive differences (post 200 ms) are likely to be related to common components in unisensory waves that are subtracted twice (e.g., attention, decision and motor).

**References**

1. Calvert GA, Thesen T (2004) Multisensory integration: methodological approaches and emerging principles in the human brain. Journal of Physiology 98: 191-205.

2. Goebel R, van Atteveldt N (2009) Multisensory functional magnetic resonance imaging: a future perspective. Experimental Brain Research 198: 153-164.

3. Laurienti PJ, Perrault TJ, Stanford TR, Wallace MT, Stein BE (2005) On the use of superadditivity as a metric for characterizing multisensory integration in functional neuroimaging studies. Experimental Brain Research 166: 289-297.

4. Fort A, Delpuech C, Pernier J, Giard MH (2002) Early auditory-visual interactions in human cortex during nonredundant target identification. Brain Research 14: 20-30.

5. Molholm S, Ritter W, Murray MM, Javitt DC, Schroeder CE, et al. (2002) Multisensory auditory-visual interactions during early sensory processing in humans: a high-density electrical mapping study. Brain Research 14: 115-128.

6. Giard MH, Peronnet F (1999) Auditory-visual integration during multimodal object recognition in humans: a behavioral and electrophysiological study. Journal of Cognitive Neuroscience 11: 473-490.
